# Adrenoceptors Modulate Cholinergic Synaptic Transmission at the Neuromuscular Junction

**DOI:** 10.3390/ijms22094611

**Published:** 2021-04-28

**Authors:** Ellya Bukharaeva, Venera Khuzakhmetova, Svetlana Dmitrieva, Andrei Tsentsevitsky

**Affiliations:** Kazan Institute of Biochemistry and Biophysics, FRC Kazan Scientific Center of RAS, 420111 Kazan, Russia; venerik87@mail.ru (V.K.); s_dmitrieva@list.ru (S.D.); atsen@list.ru (A.T.)

**Keywords:** synaptic transmission, neuromuscular junction, adrenergic drugs, adrenoreceptors, acetylcholine quantal release, acetylcholine receptor

## Abstract

Adrenoceptor activators and blockers are widely used clinically for the treatment of cardiovascular and pulmonary disorders. More recently, adrenergic agents have also been used to treat neurodegenerative diseases. Recent studies indicate a location of sympathetic varicosities in close proximity to neuromuscular junctions. The pressing question is whether there could be any effects of endo- or exogenous catecholamines on cholinergic neuromuscular transmission. It was shown that the pharmacological stimulation of adrenoceptors, as well as sympathectomy, can affect both acetylcholine release from motor nerve terminals and the functioning of postsynaptic acetylcholine receptors. In this review, we discuss the recent data regarding the effects of adrenergic drugs on neurotransmission at the neuromuscular junction. The elucidation of the molecular mechanisms by which the clinically relevant adrenomimetics and adrenoblockers regulate quantal acetylcholine release from the presynaptic nerve terminals and postsynaptic sensitivity may help in the design of highly effective and well-tolerated sympathomimetics for treating a number of neurodegenerative diseases accompanied by synaptic defects.

## 1. Introduction

The neuromuscular junction (NMJ) is the main element in the nerve stimulus transmission and the trigger for muscle contraction. Despite the long history of its study, there are still many questions about the mechanisms of the modulation of its functions in physiological and pathological states [1,2]. Two key processes provide information transfer at the NMJ: at first, a few tens of acetylcholine (ACh) quanta release from the nerve ending into the synaptic cleft after motoneuron stimulation, and secondly, there is an interaction between ACh molecules and postsynaptic ACh receptors which generates postsynaptic responses or end plate potentials (EPPs). If a sufficient number of ACh quanta are released in reply to a nerve stimulus and the EPPs amplitude reaches the critical level of muscle fiber membrane depolarization, then muscle contraction will occur. Numerous complex intracellular processes provide both the secretion of ACh quanta and the generation of muscle contraction after the activation of ACh receptors. Function disruptions of each part of the NMJ cause impaired synaptic transmission and muscle weakness [3].

In the end 19th century, it was discovered that adrenaline and noradrenaline from extracts of the suprarenal capsules increase muscle contractions [4,5]. By investigating the influence of sympathetic nerve stimulation on the skeletal muscle, Orbeli [6] and Ginetsinsky [7] found that the excitability and contractility of muscles that were fatigued due to prolonged motor nerve stimulation increased after nervus vagus excitation, which leads to the release of noradrenaline. This effect was called Orbeli-Ginetsinsky phenomenon. However, for a long time, it was not clear how catecholamines act on the NMJ and improve the skeletal muscle functions.

In the last decade, the number of publications on the effects of the sympathetic nervous system and sympathomimetics on the contractile functions of skeletal muscles has significantly increased [8,9,10,11,12,13]. Most of the works describe the action of adrenergic drugs on muscle contractions, but there is much less information regarding changes in synaptic transmission. The primary goal of this review is to discuss modern experimental data regarding the effects of adrenergic receptors activators and blockers on the quantal ACh secretion and ACh receptor function at the NMJ. This knowledge is very relevant since adrenergic compounds are widely used clinically in cardiovascular and pulmonary diseases, as well as in combination with muscle relaxants and anesthetics in surgical practice. Adrenoceptor agonists began to be offered for a treatment of wide range of neurodegenerative pathologies such as congenital myasthenic syndromes [14,15,16,17], anti-MuSK myasthenia gravis [18] and amyotrophic lateral sclerosis [19]. In recent years, experimental interest has further expanded the treatment for a wide range of muscle wasting diseases, spinal muscular atrophy and sarcopenia [13,20,21]. At the same time, it is very important to understand how adrenergic receptor activators and blockers used clinically act on the main processes of synaptic transmission in normal conditions without any pathologies. This is one of the important tasks of this review.

## 2. Activators and Blockers of Adrenergic Receptors Alter the Spontaneous ACh 

### Quantal Release at the NMJ

Without nerve stimulation, ACh quanta are released from the nerve endings spontaneously, generating miniature endplate potentials (MEPPs). The average MEPP frequency is a parameter characterizing the intensity of the secretory process in the synapse at rest [22]. There are plenty of data which indicate that adrenaline or the addition of noradrenaline to the NMJ increases the MEPP frequency [23,24,25].

There are different subtypes of the adrenoceptors for the realization of adrenaline and noradrenaline action [26]. Many experiments have been carried out with a specific agonists and antagonists. α-adrenoceptor activator phenylephrine increased the MEPP frequency and α1-adrenoceptor blocker prazosin eliminated this effect [27]. It was suggested existence of a presynaptic α1-adrenoceptor in the motor neuron terminal and suggest that modulation of transmitter release might be mediated by inositol triphosphate release, Ca^2+^ release into the cytosol and activation of a calmodulin-dependent system [27].

α2-adrenoceptor activation by clonidine and xylazine also caused an increase in MEPP frequency at the rat diaphragm NMJ, and it was eliminated by α-adrenoceptor blockers phentolamine, prazosin and yohimbine, suggesting the presence of presynaptic α1- and α2-adrenoceptors [28]. Two sympathomimetic agents used in clinical practice, salbutamol and clenbuterol, activated β2-adrenoceptors and induced the elevation of spontaneous ACh secretion in mouse preparations of peroneal nerves and lumbricalis muscles [9,29].

In contrast to these data, we observed opposite effects of the adrenergic drugs on spontaneous ACh quantal release at the mouse diaphragm NMJ at a low calcium (Ca^2+^) concentration [30]. α1- and α2-adrenoceptor activation by phenylephrine, dexmedetomidine and clonidine decreased the MEPP frequency. Previously, we observed the same effects for adrenaline and noradrenaline. The noradrenaline effect was prevented by the β-adrenoceptor blocker propranolol, whereas the adrenaline action was prevented by the α-adrenoceptor antagonist phentolamine [30,31]. The action of specific α1- and α2-adrenoceptor agonists was eliminated by blockers of α1-receptors doxazosin and α2-receptors SKF86466. β1-receptor activation by xamoterol also decreased the MEPP frequency [29,30]. The described evidences suggests the participation of various adrenoceptor subtypes in spontaneous ACh quanta secretion modulation.

The differences in the data concerning the action of the adrenergic drugs on spontaneous ACh release may be related to various experimental conditions. It was shown that sympathomimetic action on ACh secretion depends on the initial depolarization of the nerve terminal [23,24,25,27] or on extracellular calcium, sodium and magnesium ion concentration [32]. The increase in spontaneous ACh release by β2-agonists was absent in the low Ca^2+^ concentration and the TRPV1 channel activator arvanil conditions. It was suggested that sympathomimetic effects are induced by Ca^2+^ input in the nerve terminal which enhances ACh secretion. TRPV1 channel activation mediates Ca^2+^ conductance in the motor axon terminal and modulates synaptic vesicle release. When TRPV1 channels are activated by arvanil, clenbuterol does not increase the MEPP frequency [29].

The opposite effect, i.e., a decrease in spontaneous ACh secretion under adrenoceptor activation, may be due to the hyperpolarization of the nerve endings. The inhibitory effects of adrenergic drugs were blocked by β-adrenoceptor antagonists and were caused by axon membrane hyperpolarization [33]. Anderson and Harvey [34] observed an increase in the amplitude of perineural action current components, which could be a result of the hyperpolarization of the motor nerve ending in response to noradrenaline action. Wessler et al. [35] and Starke et al. [36] assumed that adrenaline and noradrenaline have opposing effects on ACh quantal release, initial facilitation and secondary inhibition, on NMJ transmission.

## 3. Sympathomimetic Effects on ACh Quantal Release Evoked by the Nerve Stimulus

In a physiological state, the NMJ operates in a rhythmic stimulation mode, and ACh quantal release evoked by motor nerve pulses induces muscle contraction. The number of synaptic vesicles released after nerve stimulus (the average quantal content) is a quantitative characteristic of the evoked ACh release. This determines the amplitude of the evoked synaptic response, i.e., the endplate potential (EPP) and muscle contraction initiation.

### 3.1. Modulation of the Number of ACh Released Quanta

Various results were obtained in the studies of the effects of adrenergic drugs on the evoked EPP amplitude and EPP quantal content. Kuba [24] and Kuba and Tomito [25] showed that noradrenaline and adrenaline increased EPP amplitude via the activation of presynaptic α-adrenoceptors. Wessler et al. [35,37,38,39] observed that α- and β-adrenoceptor stimulation mediated the increase in labeled ACh release in rat diaphragm synapses. Noradrenaline, adrenaline and phenylephrine facilitated 3H-acetylcholine evoked release. This effect was more marked when the prejunctional nicotinic receptors, mediating positive feedback modulation, were blocked by (+)-tubocurarine. Upon the application of this drug, when the 3H-acetylcholine release was reduced, α1-adrenoceptor agonists enhanced the release in a prazosin-sensitive manner. It is suggested, therefore, that endogenous catecholamines may be able to affect neuromuscular transmission through the stimulation of presynaptic α1-adrenoceptors [40].

Other works have shown that the stimulation of β-adrenoceptors enhances transmitter output from the motor nerve. It was proposed that these β-adrenoceptors are of the β1 subtype and are localized on the motor nerve endings. Endogenous catecholamines may facilitate neuromuscular transmission by the stimulation of presynaptic β-adrenoceptors [37].

Snider and Gerald [41], using a radioenzymatic assay, showed an increase in nerve-stimulated ACh release induced by noradrenaline via presynaptic α1-adrenoceptors. However, noradrenaline did not cause any change in the evoked quantal secretion in the experiments of Lim and Muir [28], whereas clonidine, phenylephrine and xylazine enhanced EPP amplitude, which was blocked by prazosin and yohimbine. In Chiou and Chang’s [42] experiments, the EPP quantal content was unaffected by clonidine, although the amplitudes of EPPs and MEPPs were markedly decreased. Our experiments on the rat soleus NMJ showed that noradrenaline did not change the number of released quanta in response to nerve stimulation. However, adrenaline at the same concentration increased evoked ACh quantal release [43]. At the diaphragm NMJ of mice in low extracellular Ca^2+^, the activation of α1-adrenoceptors by phenylephrine did not change the EPP quantal content. α2-receptor agonists clonidine and dexmedetomidine, as well as the β1-agonist xamoterol, reduced the EPP quantal content. Specific antagonists SKF and atenolol prevented the effects of activators. In contrast, procaterol, a β2-adrenoceptor agonist, slightly but significantly increased the EPP quantal content [30]. The oppositely directed action of the different types of adrenergic receptors activators may cause no effect of noradrenaline on the EPP quantal content [28,31].

Recently, Rodrigues et al. [29] also showed the involvement of presynaptic β2-adrenoceptors in the modulation of evoked ACh quantal release. β2-agonists salbutamol and clenbuterol increased evoked ACh synaptic vesicle release. This effect was mediated by presynaptic ω-agatoxin IVA-sensitive P/Q-type, ω-conotoxin GVIA-sensitive N-type Ca^2+^-channels and arvanil-sensitive TRPV1 channels.

Differences in the manifestations of the effects of adrenergic compounds on the amount of released ACh quanta are associated with the activation of the various adrenoceptor subtypes and different intracellular systems involved in the subsequent events leading to ACh release alteration. Adrenoceptor agonists are able to exert dual pharmacological effects, coupling to Gi and Gs proteins to inhibit or stimulate adenylyl cyclase activity [44]. At low agonist concentrations, α2-receptors primarily couple to Gi, whereas at high concentrations, Gs coupling dominate [45]. Starke et al. [36] supported the concept that the activation of terminal receptors coupled to G-proteins inhibits voltage-sensitive Ca^2+^-channels. L-type Ca^2+^-channels controlled by presynaptic α1-adrenoceptors at the motor nerve can be opened either directly or indirectly by second messengers (phosphoinositides, diacylglycerol, cyclic nucleotides) [35,36,44]. The mechanisms governing downstream α2-adrenoceptors activation are controversial; α2-adrenoceptor agonists can open potassium channels [44,46], inhibit voltage-dependent Ca^2+^-channels [47,48] and inhibit adenylyl cyclase [49,50,51].

Based on the available experimental data, it can be concluded that at the peripheral NMJ, there may be adrenoceptors of both α and β types, which modulate ACh secretion from motor nerve endings, increasing or decreasing it. The direction and effectiveness of such modulating effect depends on the experimental conditions (extracellular Ca^2+^ concentration, magnesium or sodium ions and the methods of muscle fiber contractions blocking), the type of muscle fibers and the functional accessory of muscles.

### 3.2. Modulation of the Kinetics of ACh Quantal Release

**Figure 1:**
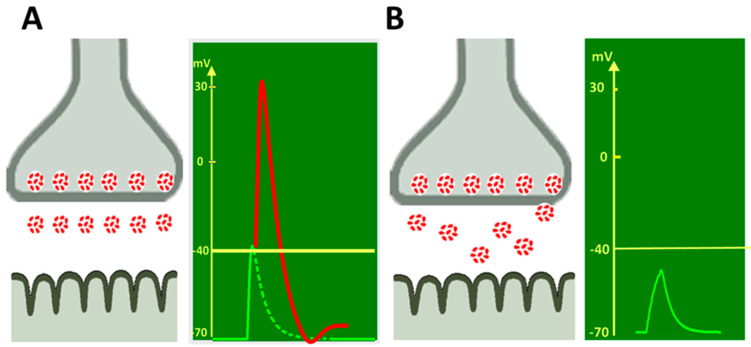
Synchronous and asynchronous ACh quanta release at NMJ. (**A**) Synchronous release of ACh quanta from nerve terminal triggers EPP in the muscle fiber with sufficient amplitude (green line), needed to generate action potential (red line) and subsequent muscle contraction. (**B**) Asynchronous release of ACh quanta triggers smaller signal (green line) in the muscle fiber, insufficient to cause action potential generation and muscle fiber contraction.

It is generally accepted that in addition to quantal content, another important characteristic of evoked neurotransmitter release is the kinetics or time course of ACh quantal secretion [52,53,54]. The release of several tens of ACh quanta in response to a single nerve stimulus does not occur simultaneously [52,53,55]. The asynchrony of individual quanta secretion is manifested by the dispersion of real synaptic delays of uni-quantal EPPs recorded in low Ca^2+^/high Mg^2+^ conditions [52,55] or by a greater increase in the rise time of multi-quantal EPP compared to the MEPP rise time [56,57]. More synchronous release of quanta leads to the EPP amplitude increasing and thus facilitates synaptic transmission [58] (Figure 1).

**Figure 2:**
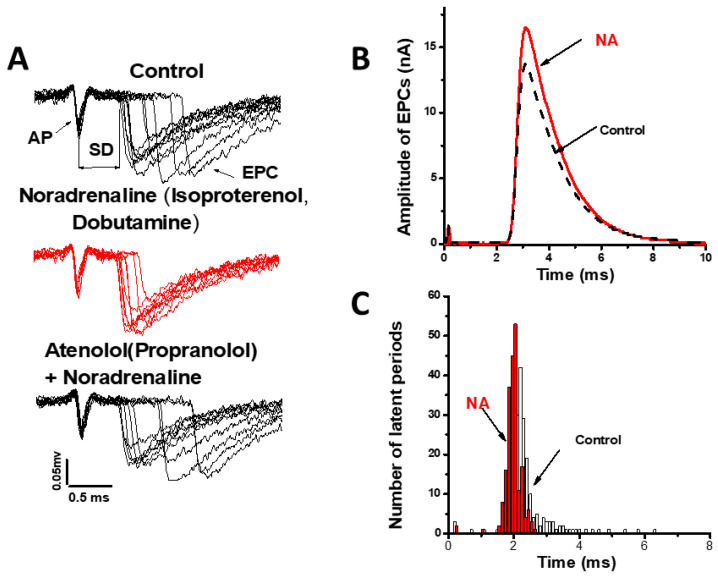
Noradrenaline and β1 adrenoceptors agonists effects at frog NMJ. (**A**) decrease of the synaptic delays (SD) fluctuations of extracellular uni-quantal EPCs (end plate currents) in the presence of noradrenaline or agonists of β1 adrenoceptors (middle panel), synaptic delays after noradrenaline addition in presence β1 adrenoceptor blockers; (**B**) intracellular multi-quantal EPC has higher amplitude after noradrenaline application without increase of EPC quantal content; (**C**) histograms of synaptic delays distributions in control and after noradrenaline application. AP—axon action potential; NA—noradrenaline.

We have previously shown that noradrenaline synchronizes evoked ACh quantal release without quantal content change at the frog NMJ [59] (Figure 2).

**Figure 3:**
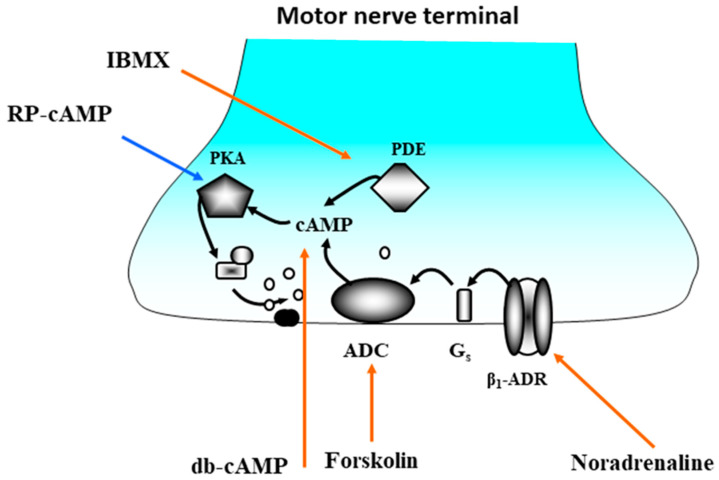
β1-adrenoceptor activation by noradrenaline causes the synchronization of ACh quanta release at frog NMJ by participation of Gs protein, activation of adenylyl cyclase (ADC), increase in cAMP production and proteinkinase A (PKA) activation. Red arrows show synchronizing action of noradrenaline, activator of ADC forskoline, db-cAMP—permeable derivative of cAMP, phosphodiesterase (PDE) inhibitor isobutylmethylxanthine (IBMX). Stimulation-evoked EPCs with long release latencies were eliminated when the intracellular cAMP was increased by β1-adrenoceptor activation by noradrenaline, by the permeable analogue db-cAMP, by activation of adenylyl cyclase or by inhibition of cAMP hydrolysis. Protein kinase A is a target of this regulation, since a specific inhibitor, Rp-cAMP, prevents the action of cAMP and noradrenaline [60].

Pharmacological analysis with specific agonists and antagonists of the adrenoceptors showed that the synchronizing effect of noradrenaline is due to the activation of presynaptic β1-adrenergic receptors. The sequence of intracellular events that occur after the activation of presynaptic β1-adrenoceptors by noradrenaline includes the activation of adenylyl cyclase, an increase in the intracellular level of cAMP, activation of protein kinase A and phosphorylation of synaptic proteins, leading to an acceleration of exocytosis of the contents of synaptic vesicles [60] (Figure 3).

It was shown that the synchronizing action of noradrenaline leads to an increase in the amplitude of the multiquantal current of the end plate in the “fatigued” muscle and underlies the Orbeli–Ginetsinsky phenomenon—an increase in muscle contractions upon stimulation of the sympathetic nerve [6,7]. Therefore, the synchronization of ACh secretion may be a mechanism for the facilitating action of the sympathomimetics [58].

Quantal secretion asynchrony is determined by various factors, such as Ca^2+^ entry into the nerve terminal, intracellular Ca^2+^ buffer activity, exocytosis proteins and intracellular enzyme systems [55,61]. It was shown previously that the degree of secretion synchronization at the mouse NMJ depends on Ca^2+^ entry into the nerve ending. The synchrony of quantal secretion increased with the growth of extracellular Ca^2+^ [55]. This relationship is since intracellular Ca^2+^ determines the rate of Ca^2+^ activated vesicular fusion, so that higher Ca^2+^ accelerates the rate of the release reaction and induces short synaptic delays. [55,62]. Recently, we investigated the effects of adrenergic drugs on the kinetics of ACh quantal release at mammal NMJs. In our experiments, adrenaline increased spontaneous and evoked quantal ACh release at the NMJ of the hindlimb soleus muscle and synchronized evoked ACh quantal secretion [43]. We suggested that the facilitation of the synaptic transmission by adrenaline is due to the increased Ca^2+^ entering the axoplasm. There is evidence that sympathomimetics affect the activity of voltage gated Ca^2+^ channels [29,39,62], which may be the mechanism of the effect of adrenoreceptor activation on the kinetics of secretion.

However, at the mouse diaphragm NMJ, the opposite desynchronization effect of noradrenaline was observed, and both α- and β-receptors were involved in this action [31]. In contrast to the observations at frog and soleus muscle NMJ, data obtained demonstrate that the activation of α2-adrenoceptors by dexmedetomidine and β2-adrenoceptors by procaterol increased the ACh quantal secretion asynchrony at mouse diaphragm. These effects were eliminated by α2- and β2-adrenoceptor blockers [30,31].

Thus, the analysis of the effects of adrenergic drugs on the ACh quantal release at the NMJ showed diverse modulation of the spontaneous and evoked ACh quantal release from motor nerve endings in response to the activation of various subtypes of adrenergic receptors (Table 1). Obviously, further research is required to understand the features and mechanisms of adrenergic drugs action on the ACh quantal secretion.

## 4. Effects of Adrenergic Drugs on the Postsynaptic Nicotinic Receptors at the NMJ

Adrenergic drugs act not only on the release of ACh quanta. There are numerous data regarding the state of ACh receptors changing on the postsynaptic membrane of muscle fibers in response to catecholamine action. As mentioned above, the interaction of ACh molecules with their postsynaptic receptors determines the amplitude of postsynaptic potential and the onset of muscle contraction. For the estimation of ACh receptor functions, analysis of the amplitude-temporal parameters of miniature end plate current (MEPC) was used. The MEPC amplitude is affected by the number of ACh molecules per quanta and by the single channel conductance. It was shown that albuterol, ephedrine and pseudoephedrine acted as a fast-acting channel blocker of ACh receptors [63,64]. Ephedrine decreased MEPP amplitude. It reduced the channel conductance but had no effect on the channel’s open state duration [64]. The ACh receptor channel blocking effect of ephedrine and pseudoephedrine reduced the synaptic transmission that occurs in the slow-channel myasthenic syndromes or endplate acetylcholinesterase deficiency [65]. We did not observe significant changes of the MEPP amplitude–temporal parameters in response to phenylephrine, doxazosin, dexmedetomidine or clonidine application at the mouse diaphragm NMJ. This indicates the absence of postsynaptic α1- and α2-adrenoceptors at the NMJ. β1-receptor agonist xamoterol and its antagonist atenolol also did not change the MEPP parameters. Our data showed the absence of postsynaptic β1-adrenoceptor influence on the endplate electrogenesis [30].

In contrast, β2-agonist salbutamol induced the significant improvements in several postsynaptic morphological defects including increased synaptic area, density of ACh receptors, extension of postjunctional folds and significant increases in time to peak, half-decay time and the duration of MEPP at the mouse NMJ with the ColQ myasthenic syndrome model [8]. These effects are mainly manifested at the postsynaptic membrane and can lead to enhanced synaptic transmission. Moreover, salbutamol induces a significant increase in the number of ACh receptor clusters by affecting proteins located at the NMJ [9,66,67]. The sympathetic nervous system regulates postsynaptic ACh receptors stability and clustering through the action of endogenous catecholamines on the sarcoplasm proteins Gαi2-Hdac4-myogenin-MuRF1 [9].

The data analysis of the postsynaptic action of adrenergic drugs suggests that mainly β2-adrenoceptor agonists regulate the nicotinic ionotropic ACh receptor state at the NMJ. Here, we describe the effects of adrenergic drugs on ACh receptors in the NMJ postsynaptic area. However, there are many data regarding the effects of muscle β2-adrenoceptor activation on the intracellular processes in muscle fibers on extrasynaptic zones [68,69]. These results require separate consideration.

## 5. Morphological Evidence of the Existence of Adrenoceptors at the NMJ

The data described above suggest the presence of adrenoceptors at the NMJ. Western blotting was used to identify different subtypes of adrenoceptors in the mouse diaphragm. Analysis revealed the presence of α1A, α1B, α2A, α2B, α2C and β1 subtypes of adrenoceptors [30]. Presynaptic α2-adrenoceptors are involved in the downregulation of noradrenaline release from sympathetic axon terminals near the NMJ activating Gαi/s protein and inhibiting voltage-gated N-type Ca^2+^ channels [8,29,66]. The postsynaptic effects of β2 adrenergic drugs indicate the presence of this type of receptor in the endplate area. Co-localization of β2-adrenoceptors with ACh receptors was shown by immunostaining method in mouse gastrocnemius and extensor digitorum longus muscles [8]. This is consistent with the statement that β2-adrenoceptors are the dominant subtype in the skeletal muscle of rodents, constituting 80–90% of the total number of adrenergic receptors [68,69,70].

Presynaptic action of adrenergic drugs on the ACh release suggests the existence of adrenoceptors at the motor nerve ending. Sympathetic innervation of the NMJs in hindlimb, diaphragm, and levator auris longus muscles is extensive and, in the extensor digitorum longus muscle, increases until adulthood [71]. Immunofluorescence co-expression analysis of immunolabeled adrenoceptors with choline acetyltransferase-, tyrosine hydroxylase- or calcitonin gene-related peptide immunoreactive axons showed that α2B-adrenoceptors were found mainly in sympathetic neurons, and β1 type adrenoceptors in sympathetic and motor neurons [3,29,67,72]. It was suggested that presynaptic β-adrenoceptors presumably mediate catecholamine and sympathomimetic effects on motoneuron axonal Ca^2+^ channels during NMJ activation [8,9,29].

## 6. Effects of the Endogenous Catecholamines on Synaptic Transmission

An intriguing question is whether endogenous adrenaline and noradrenaline are able to affect neuromuscular transmission. Studies using analysis of the localization of NMJ and sympathetic innervation and the elimination of sympathetic influences on the synapse give a positive response.

At the early 20th century, it was found that sympathetic nerve fibers have close contacts with the striated muscles fibers [73]. Later, with the development of immunofluorescence techniques and confocal microscopy, the co-localization of tyrosine hydroxylaze, a marker of sympathetic neurons, and the endplate of the muscle fiber was directly demonstrated [9,67,71].

It was presented evidences that the NMJs of various muscles, such as tibialis anterior, soleus, levator auris longus and diaphragm are directly innervated by sympathetic neurons [11,12,13]; the innervation starts before birth and actively continues during postnatal development [71].

The influence of adrenoceptor blockers on the synaptic transmission was also observed. α2-adrenoceptor inhibition by SKF 86466 and β2-adrenoceptors by ICI 118,551 diminished spontaneously and evoked ACh quantal release. β1-adrenoceptor antagonist atenolol synchronized the secretory process [30]. These observations can indicate the action of the endogenous noradrenaline at the NMJ.

The effects of sympathectomy caused a significant reduction in nerve-evoked muscle contraction [71] and the decrease in ACh quantal release [29], demonstrating the possibility of endogenous catecholamine effects on the synaptic function. It was shown that the sympathetic nervous system controls the organization and function of pre- and postsynaptic cells regulating the expression of genes encoding neurotrophins and motor axon neurofilament phosphorylation. The ablation of the sympathetic innervation leads to the upregulation of several important postsynaptic proteins (LRP4, MuSK, DOK7, MuRF1), muscle atrophy and the downregulation of postsynaptic ACh receptors [9,29,67]. Sympathomimetic agents reestablish the muscle action potentials disrupted by chemical ablation of sympathetic innervation [29,71]. Direct stimulation of sympathetic neurons enhances neuromuscular transmission in young mice by catecholamine released from sympathetic axons that act on β-adrenoceptors expressed in the motoneuron [71]. In contrast, sympathectomy effects on neuromuscular transmission involve disorganization of the cytoskeleton in motor axons, myelination defects, decreases in axon diameter and fiber-type specific reductions in the muscle cross-sectional area [8,9,29].

Thus, it can be concluded that sympathetic innervation provides the presence of endogenous catecholamines near the NMJ and is involved in the modulation of synaptic transmission.

## 7. Progress and Perspectives

**Figure 4:**
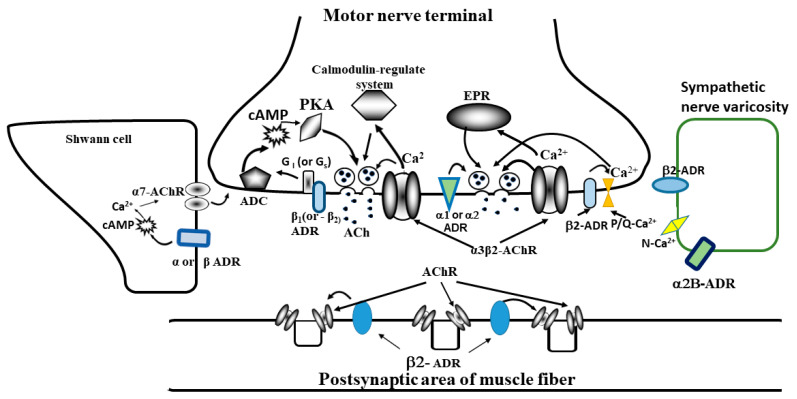
Hypothetical scheme of mammalian NMJ with the sympathetic innervation. ACh-quanta of acetylcholine; ADR—adrenoceptors various types (α1, α2, α2B; β1, β2;); AChR—postsynaptic ACh receptors; α3β2-AChR—neuronal presynaptic ACh receptors; α7-AChR—neuronal AChR receptors in Schwann cell; ADC—adenylate cyclase; cAMP—cyclic adenosine monophosphate; PKA—protein kinase A; EPR—endoplasmic reticulum; Gi or Gs—G-protein; P/Q-Ca^2+^—voltage dependent calcium channel P/Q type; N-Ca^2+^—voltage dependent calcium channel N type. Orange arrows show possible action the adrenoceptor’s activation.

Pharmacological analysis of the effects of adrenergic drugs, specific for various adrenoceptors subtypes, showed that the activation and blockade of various subtypes of α- and β-adrenoceptors modulate ACh quantal release from motor nerve terminals and postsynaptic ACh receptor properties; thus, they can affect the efficiency of synaptic excitation transmission at the NMJ. According to the latest experimental data, α1- and α2 (subtypes B)-adrenoceptors were found in the presynaptic zones of motor nerve and sympathetic nerve endings. They regulate noradrenaline release from sympathetic varicosities and spontaneous ACh secretion from motor nerve endings. β1-adrenoceptors can be localized on sympathetic axons and at the same time on motoneuron endings, whereas β2-adrenoceptors are mainly on the muscle fiber membrane. Schwann cells surrounding the neuromuscular synapse may be involved in adrenergic modulation of synaptic transmission. Indeed, adrenaline and noradrenaline application induced the intracellular Ca concentration increase, raised intracellular cyclic AMP in Schwann cell [74] and, as a consequence, the release of some gliotransmitters adenosine [75] or presumably gamma-aminobutyric acid [76,77], which affect the secretion of ACh [78]. These data suggest that α- or β-adrenoceptors are present on the Schwann cells membrane [79].Endogenous catecholamines and exogenous adrenergic drugs alter the cholinergic synaptic transmission by activating various subtypes of adrenergic receptors, as well as can act on the membrane of motor nerves and on the extrasynaptic membrane of muscle fibers. Based on the available experimental data, it is possible to propose a hypothetical scheme of NMJ with the sympathetic innervation (Figure 4).

We show all the components (pre- and postsynaptic ACh receptors, various subtypes of adrenoceptors, calcium channels) which may participate или capable to participate in the adrenergic modulation of the NMJ function. The precise molecular mechanisms by which adrenoceptors modulate ACh quantal release and nicotinic ACh receptor sensitivity is not yet definitively known and requires further study. The main hypotheses suggest that the influence of adrenergic compounds is associated with a change in the Ca^2+^ input to the motor nerve endings through the potential-sensitive Ca^2+^ channels and/or with a change in the state of different G-proteins and the activation of the adenylyl cyclase pathway. The effects on postsynaptic ACh receptors are mediated by changes in the state of proteins involved in ACh receptor clustering.

The interest in research on the interaction of adrenergic and cholinergic receptor systems has grown significantly over the past few years. This is due to the fact that new data are emerging that indicate the important role of sympathetic innervation in providing a better functional state of skeletal muscles during aging and some pathological conditions accompanied by synaptic defects. The therapeutic pre- and postsynaptic benefits of β2 adrenoceptor agonists (salbutamol and ephedrine) have been reported in a MuSK myasthenia gravis animal model and in patients with autoimmune myasthenia Gravis and various types of congenital myasthenic syndromes.

Delbone et al. [13] presented a large review of the data obtained from analyzing the interaction of the sympathetic and motor nervous systems and described the importance of modulating the functional state of muscles with endogenous catecholamines and their possible role in the prevention of sarcopenia and muscle weakness with aging. This is very significant for the development of new treatments for muscle weakness in aging and diseases with synaptic defects.

The results reviewed confirm that the sympathetic nervous system has strong and diverse effects on synaptic transmission at the NMJ. All these findings demonstrate a novel mechanism for modulating synaptic transmission based on the interaction between the motor and sympathetic nervous system at the NMJ and stimulate researchers to further study the effects of sympathetic innervation on processes in the somatic peripheral nervous system.

## Figures and Tables

**Table 1:** Effects of agonists of various types of adrenergic receptors on the quantal ACh release.

ADR	Spontaneous ACh Release		Evoked ACh Release			
			Quantal Content		Secretion Kinetics	
	Inhibition	Facilitation	Inhibition	Facilitation	Synchronization	Desynchronization
α	Adrenaline * Noradrenaline * Phenylephrine * Dexmedetomidine * Clonidine * [30,31,49]	Noradrenaline [23,24] Adrenaline [25]		Noradrenaline Adrenaline α-methylnor-adrenaline [32,33]	Adrenaline * [30,31]	Noradrenaline * [30,31]
β		Noradrenaline [24]		Isoprenaline Noradrenaline [23,24,32,33,35,37]		
α1	Phenylephrine [31]	Phenylephrine ^K^ [27,41]		Phenylephrine [33,38,40]	Adrenaline * [30,31]	
α2	Dexmedetomidine * Clonidine * [30]	Clonidine Xylazine [28]	Clonidine * Dexmedetomidine * [30]		Adrenaline * [31,43]	Dexmedetomidine [30] *
β1	Xamoterol * [30]	Isoproterenol [25]	Xamoterol * [30]	Isoprenaline [27,37,39]	Noradrenaline [59]	
β2		Salbutamol Clenbuterol [9,29]		Salbutamol Clenbuterol Procaterol * [9,29]		Procaterol * [30]

Adrenoceptor (ADR) agonists affecting the parameters of the quantal ACh release, acting on different subtypes receptor are presented. Noradrenaline and adrenaline can interact with both α and β receptors, therefore their effects are related α (or β) receptors if they are eliminated by α (or β) blockers respectively. Different experimental condition: *—Low extracellular [Ca^2+^]_o_ concentration; ^K^—High extracellular potassium concentration; [30]—reference number.

## Data Availability

Not applicable.
